# Accuracy of 16/18G core needle biopsy for ultrasound-visible breast lesions

**DOI:** 10.1186/1477-7819-12-7

**Published:** 2014-01-08

**Authors:** Jie-Ying Zhou, Jie Tang, Zhi-Li Wang, Fa-Qin Lv, Yu-Kun Luo, Hong-Zhen Qin, Mei Liu

**Affiliations:** 1 Department of Ultrasound, Chinese People’s Liberation Army General Hospital, 28 Fuxing Road, Beijing 100853, China; 2 Department of Breast Surgery, Chinese People’s Liberation Army General Hospital, 28 Fuxing Road, Beijing 100853, China; 3 Department of Pathology, Chinese People’s Liberation Army General Hospital, 28 Fuxing Road, Beijing 100853, China

**Keywords:** Breast, Beast cancer, Core needle biopsy, Surgical excision, Ultrasound

## Abstract

**Background:**

To assess the accuracy of ultrasound-guided 16G or 18G core needle biopsy (CNB) for ultrasound-visible breast lesions, and to analyze the effects of lesion features.

**Methods:**

Between July 2005 and July 2012, 4,453 ultrasound-detected breast lesions underwent ultrasound-guided CNB and were retrospectively reviewed. Surgical excision was performed for 955 lesions (566 with 16G CNB and 389 with 18G CNB) which constitute the basis of the study. Histological findings were compared between the ultrasound-guided CNB and the surgical excision to determine sensitivity, false-negative rate, agreement rate, and underestimation rate, according to different lesion features.

**Results:**

Final pathological results were malignant in 84.1% (invasive carcinoma, ductal carcinoma in situ, lymphoma, and metastases), high-risk in 8.4% (atypical lesions, papillary lesions, and phyllodes tumors), and benign in 7.5%. False-negative rates were 1.4% for 16G and 18G CNB. Agreement rates between histological findings of CNB and surgery were 92.4% for 16G and 92.8% for 18G CNB. Overall underestimate rates (high-risk CNB becoming malignant on surgery and ductal carcinoma in situ becoming invasive carcinoma) were 47.4% for 16G and 48.9% for 18G CNB. Agreements were better for mass lesions (16G: 92.7%; 18G: 93.7%) than for non-mass lesions (16G, 85.7%; 18G, 78.3%) (*P* <0.01). For mass lesions with a diameter ≤10 mm, the agreement rates (16G, 83.3%; 18G, 86.7%) were lower (*P* <0.01).

**Conclusions:**

Ultrasound-guided 16G and 18G CNB are accurate for evaluating ultrasound-visible breast mass lesions with a diameter >10 mm.

## Background

Percutaneous ultrasound- and stereotactic-guided breast core needle biopsy (CNB) is widely used as a reliable alternative to surgical biopsy to obtain a histological diagnosis for imagery-visible suspicious breast lesions [1-3]. Stereotactic-guided percutaneous breast biopsy is mostly used for micro-calcifications, while ultrasound-guided biopsy is mostly used for masses and architectural distortions. Previous studies showed that stereotactic-guided CNB using a large needle gauge provided an accurate diagnosis [4,5].

However, the accuracy of using 16G and 18G needles for ultrasound-guided CNB, especially for ultrasound-visible mass lesions, have not been fully assessed. Few distinctions are made between stereotactic and ultrasound-guidance CNB when needle size is discussed [6]. Little information is currently available about needle size selection for ultrasound-guided biopsies of lesions with different imagery features, and most of this information comes from non-breast lesions [7], or from studies that used 14G needles only [8-18]. Therefore, one may raise the question about whether ultrasound-guided CNB with smaller needles, such as 16G and 18G, can have diagnostic value for ultrasound-visible lesions, and if there is any difference in the accuracy of biopsies of breast lesions with different imaging features.

In this article, we present a large series of breast lesions that underwent ultrasound-guided 16G or 18G CNB followed by subsequent surgical excision. We hypothesized that smaller gauge CNB provides results that are usable in a routine clinical setting. The objectives were to assess the accuracy of this technique for ultrasound-visible breast lesions, and to analyze the effects of lesion features. The results of this study could allow the use of needles that are less expensive and less traumatic for the patient, without impairing the accuracy of the biopsy.

## Methods

### Patients

From July 2005 to July 2012, 4,453 consecutive ultrasound-detected breast lesions were biopsied in our department. Out of these 4,453 biopsies, 2,646 were performed using 16G CNB, and 1,807 were performed using 18G CNB. Inclusion criteria were: i) ultrasound-visible breast lesion; ii) 16G or 18G CNB; iii) subsequent surgery to remove the lesion; and iv) a complete dataset. Exclusion criteria were: i) previous surgery of the target lesion; ii) stereotactic biopsy; or iii) any intervention performed on the target lesion before ultrasound-guided CNB. A total of 934 patients (928 women, 6 men) with 955 breast lesions were therefore included in the present study.

This study was approved by the ethical committee of our hospital and complied with the Health Insurance Portability and Account Ability Act. According to our hospital guidelines, informed consent for biopsy and use of related data for future study was obtained from each patient before the examination.

### Lesions

Breast Imaging Reporting and Data System (BI-RADS) categories are routinely used for reporting lesions at our department. Lesions categorized as BI-RADS 4 or 5 usually undergo biopsy. Lesions categorized as BI-RADS 3 may also undergo biopsy if the lesions are evolving during follow-up or according to patients’ and/or surgeons’ will. All lesions were: i) ultrasound-visible solid lesions; ii) lesions with a solid component on ultrasound; or 3) non-mass lesions visible on ultrasound. An ultrasound-visible “mass lesion” is a space-occupying lesion, while a “non-mass lesion” is visible as an irregular hypoechoic area with an indistinct margin on multiple different ultrasound images (heterogeneous hypoechoic area, ill-defined lobulated mass, or an enlarged duct) [19].

### Biopsy procedure

At our department, an ultrasound-guided biopsy is initially performed on ultrasound-visible breast lesions regardless of their palpability; 16G and 18G CNB are routinely used and the choice is predominantly determined by the radiologist’s preference. When patients have poor blood dyscrasia or are under anticoagulant therapy, an 18G CNB is strongly recommended. All biopsies were performed by a team of 9 radiologists with either fellowship training or an extensive clinical image and interventional experience of at least 5 years in breast imaging and biopsy.

Ultrasound examinations were performed using a Sequoia 512 scanner (Siemens-Acuson, Mountain View, CA, USA) equipped with a 15L8w linear-array transducer, and an iU22 scanner (Philips Ultrasound, Bothell, WA, USA) with a L12-5 linear-array transducer. Biopsies were performed with a 16G or 18G core needle (CR Bard, Covington, GA, USA) with 15- or 22-mm throw. The choice of needle excursion (short throw: 15 mm or long throw: 22 mm) was based on the lesion size, its position, and the thickness of glandular parenchyma. For lesions smaller than 15 mm or near the pectoralis major muscle or if the patient’s glandular parenchyma was very thin, the short throw device was used; on the contrary, for lesions larger than 15 mm or without special location, the long throw device was routinely used.

Two or three core samples were obtained in most of the lesions, while two to six core samples were harvested when the lesion had indistinct margins, such as non-mass lesions. The appearance and behavior of the formalin-fixed core samples were examined during the procedure to confirm that the targeted lesion was adequately sampled.

Post-fire needle position verification was routinely performed in all biopsies. The punctures were compressed for 5–10 min to control bleeding.

### Post-biopsy management

CNB pathological examinations were performed by a dedicated breast pathologist, and the results were categorized as malignant, high-risk, or benign. Malignant results included invasive carcinoma, ductal carcinoma in situ (DCIS), lymphoma, and metastases. High-risk results included atypical ductal hyperplasia (ADH), atypical lobular hyperplasia (ALH), lobular carcinoma in situ (LCIS), papillary lesions (intra-ductal papilloma and papilloma with atypia), and phyllodes tumors. All other lesions were categorized as benign findings [16]. Occasionally, when the findings were unclear, a group of pathologists examined the specimens and the final histopathological results were documented as the highest risk lesion.

The radio-pathological concordance was performed between CNB results and imaging findings for each case. If the CNB yielded malignant lesions, the patient underwent the respective surgery. In high-risk cases on CNB, indeterminate cases, or radio-pathological discordance, surgical excision was performed. If the CNB yielded benign results concordant with imaging, long-term follow-up was recommended. However, some concordant benign lesions underwent surgical excision at the patient’s request.

### Data analysis

The diagnostic accuracy of ultrasound-guided CNB was assessed based on the gold standard of the histopathological results after surgical excision. Sensitivities, false-negative rates, agreement rates, high-risk underestimation rates, and DCIS underestimation rates were calculated for ultrasound-guided CNB.

The high-risk underestimation rate was defined as the proportion of lesions diagnosed as high-risk by CNB that were upgraded to malignancy after surgical excision. The DCIS underestimation rate was defined as the proportion of lesions diagnosed as DCIS by CNB but upgraded to invasive cancer after surgical excision. The false-negative rate was defined as the proportion of all breast cancers at surgery with a benign diagnosis on CNB. The complete sensitivity was defined as the false-negative rate subtracted from one. The absolute sensitivity rate was defined as the proportion of malignancies that were identified by ultrasound-guided CNB. The agreement rate was defined as the proportion of lesions that were not classified as DCIS underestimation, high-risk underestimation, or false-negative diagnosis. For clinically-relevant purposes, a lesion with a benign diagnosis on CNB which was then upgraded to high-risk after excision, was reclassified as being in agreement because no clinical consequences would result [20].

For the false-negative rates, sensitivities, underestimation rates, and agreement rates of the ultrasound-guided CNB, the type of imagery lesion (mass or non-mass), lesion size (≤5, 6–10, 11–20, 21–50, and >50 mm), needle gauge (16G or 18G), and calcifications (with or without) were assessed.

All analyses were performed using SPSS 18.0, standard version (SPSS Inc., Chicago, IL, USA). Continuous variables were expressed as mean ± SD. Student’s *t*-test was used to analyze continuous variables. The χ^2^ test, Fisher’s exact test, or Kruskal-Wallis test were used for univariate comparisons. *P* values <0.01 were considered statistically significant.

## Results

Patients’ mean age was 48.9 ± 11.4 years (range: 15–85) for 16G CNB and 49.8 ± 11.3 years (range: 16–87) for 18G CNB. Among all lesions, 885 lesions were BI-RADS 4 or 5, and 70 were BI-RADS 3. The mean lesion size was 22 ± 12 mm (range: 3–100) in the 16G group and 23 ± 13 mm (range: 4–83) in the 18G group. The mean number of cores for 16G and 18G CNB were 2.8 ± 0.5 (range: 2–6) and 2.8 ± 0.5 (range 2–6), respectively. There were no differences in the breast lesion characteristics between the two gauges (Table 1).

**Table 1:** Characteristics of ultrasound-guided 16G/18G CNB of breast lesions

**Variables**	**Total (n = 955)**	**16G (n = 566)**	**18G (n = 389)**	P*
Age group				0.796
<40	166	102	64	
40–50	382	234	148	
50–60	235	133	102	
60–70	119	62	57	
≥70	53	35	18	
BI-RADS^#^				0.459
3	70	42	28	
4	568	345	223	
5	317	179	138	
Lesions size (mm)				0.065
≤10	133	72	61	
11–20	387	235	152	
21–50	404	244	160	
>50	31	15	16	
Calcifications				0.056
Present	319	176	143	
Absent	636	390	246	
Lesions type				0.082
Mass	911	545	366	
Non-mass	44	21	23	

*According to unpaired *t*-test, χ^2^ test, or Fisher’s exact test, as appropriate.

BI-RADS: breast imaging reporting and data analysis.

^#^3: Probably benign lesions; 4: Suspicious of malignancy (possibility 3%–94%); 5: Highly suggestive of malignancy (>95%).

Pathological examination of the CNB revealed that malignant lesions accounted for 84.1% (n = 803) of CNB, high-risk lesions accounted for 8.4% (n = 80), and benign lesions accounted for 7.5% (n = 72) (Table 2).

**Table 2:** Histopathological findings of breast ultrasound -guided CNB

**Pathology findings (n = 955)**	**16G (n)**	**18G (n)**
Benign	39	33
Breast normal tissue	3	1
Fibroadenoma	9	9
Chronic inflammation and fat necrosis	11	11
Adenosis	16	12
High-risk	50	30
ADH	13	12
ALH	0	0
LCIS	0	0
Papillary lesion	18	6
Atypical papillary lesion	4	5
Phyllodes	5	1
Radial scar	2	1
Not specified	8	5
Malignant	477	326
DCIS	26	17
IDC	405	282
ILC	7	4
Mucinous Ca	10	11
Papillary Ca	4	3
Tubular Ca	2	1
Medullary Ca	3	0
Lymphoma	1	1
Minimal invasion	1	0
Invasive carcinoma not specified	18	7
Total	566	389

ADH: Atypical ductal hyperplasia; ALH: Atypical lobular hyperplasia; DCIS: Ductal carcinoma in situ; IDC: Invasive ductal carcinoma; ILC: Invasive lobular carcinoma; LCIS: Lobular carcinoma in situ; Ca: Carcinoma.

False-negative rates, sensitivities, underestimation rates, and agreement rates of 16G and 18G CNB compared with the final surgical histological results were calculated according to the CNB gauge, lesion type, lesion size, and calcifications (Table 3). False-negative rates were 1.4% for 16G and 18G CNB. Agreement rates between histological findings of CNB and surgery were 92.4% for 16G and 92.8% for 18G CNB, and there was no difference between the two gauges (*P* >0.01). However, there was a risk of underestimation with 16G and 18G CNB. The high-risk underestimation rates were 24/50 (48.0%) for 16G CNB and 16/30 (53.3%) for 18G CNB. The DCIS underestimation rates were 12/26 (46.2%) for 16G CNB and 7/17 (41.2%) for 18G CNB. There were no statistical differences between the two gauges.

**Table 3:** Breast CNB accuracy according to gauge, lesion type, lesion size and calcifications

	**False-negative**	**Underestimation**	**Complete sensitivity**	**Absolute sensitivity**	**Agreement**
	**n (%)**	**n (%)**	**%**	**n (%)**	**n (%)**
18G (n = 389)					
Mass	4/327 (1.2)	19/38 (50)	98.8	308/327 (94.2)	343/366 (93.7)
Non-mass	1/19 (5.3)	4/9 (44.4)	94.7	17/19 (89.5)	18/23 (78.3)
Mass Size (mm)					
≤10	2/47 (4.3)	6/18 (33.3)	95.7	39/47 (83.0)	52/60 (86.7)
11–20	1/134 (0.7)	7/10 (70)	99.3	129/134 (96.3)	141/149 (94.6)
21–50	1/137 (0.7)	6/10 (60)	99.3	131/137 (95.6)	139/146 (95.2)
>50	0/9 (0)	0/0 (0)	100	9/9 (100)	11/11 (100)
Calcifications					
Present	2/133 (1.5)	6/15 (40)	98.5	129/133 (97.0)	135/143 (94.4)
Absent	3/213 (1.4)	17/32 (53)	98.6	196/213 (92.0)	226/246 (91.9)
Overall	5/346 (1.4)	23/47 (48.9)	98.6	325/346 (93.9)	361/389 (92.8)
16G (n = 566)					
Mass	7/488 (1.4)	33/68 (48.5)	98.6	460/488 (94.3)	505/545 (92.7)
Non-mass	0/17 (0)	3/8 (37.5)	100	14/17 (82.4)	18/21 (85.7)
Mass Size (mm)					
≤10	2/58 (3.4)	10/19 (52.6)	96.6	50/58 (86.2)	60/72 (83.3)
11–20	3/208 (1.4)	14/24 (58.3)	98.6	198/208 (95.2)	215/232 (92.7)
21–50	2/213 (0.9)	9/24 (37.5)	99.1	203/213 (95.3)	219/230 (95.2)
>50	0/9 (0)	0/1 (0)	100	9/9 (100)	11/11 (100)
Calcifications					
Present	2/166 (1.2)	6/18 (33.3)	98.8	162/166 (97.6)	168/176 (95.5)
Absent	5/339 (1.5)	30/58 (51.7)	98.5	312/339 (92.0)	355/390 (91.0)
Overall	7/505 (1.4)	36/76 (47.4)	98.6	474/505 (93.9)	523/566 (92.4)

For both 16G and 18G CNB, the absolute sensitivities and agreement rates were better for mass lesions than for non-mass lesions, reaching statistical significance in 18G CNB (absolute sensitivity: mass: 94.2% vs. non-mass: 89.5%; agreement: mass: 93.7% vs. non-mass: 78.3%; all *P* <0.01). Results showed a significant trend toward a better agreement of CNB with increasing mass size. Indeed, false-negative rates of CNB were the highest and agreement rates were the lowest when mass size was ≤10 mm (accounting for 13.9% of CNB in the present study). In addition, although lesions with calcification had higher agreement rates (18G: 94.4 vs. 91.9%; 16G: 95.5 vs. 91.0%) and lower underestimation rates (18G: 40.0 vs. 53.0%; 16G: 33.3 vs. 51.7%) than lesions without calcification (Table 3), there were no significant differences between the two groups.

## Discussion

Minimally invasive percutaneous CNB is used for ultrasound-visible breast lesions, but no study has previously assessed the accuracy of CNB using 16G or 18G needles. The objective was then to assess the accuracy of this technique for ultrasound-visible breast lesions, and to analyze the effects of lesions’ image features. Results showed that false-negative rates were low for 16G and 18G CNB, and that agreement rates between histological findings of CNB and surgery were >92% for 16G and 18G CNB. Agreements were better for mass lesions than for non-mass lesions. Agreement rates were lower for mass lesions with a size ≤10 mm.

Ultrasound-guided CNB has become the first biopsy choice for ultrasound-visible lesions [21]. It offers several advantages over stereotactic guidance, including no radiation, full real-time control of the needle position, and patients’ comfort without breast compression. It is also more flexible for biopsy of multiple lesions or in difficult places (such as the axilla or near the nipple) [22]. Previous studies showed that ultrasound-guided CNB had a high accuracy when using 14G needles [2,10,13,16,20-22]. However, very little information is currently available about how these results may vary when the biopsies are performed using smaller size needles, and most of these results are from non-breast studies [7].

China has different ethnic populations and different medical treatment situations compared with most Western countries. In addition, Asian women are observed to have smaller breasts and denser mammary parenchyma than Caucasian women [23]. Due to the magnitude of breast cancer risk factors, genetic and/or environmental discrepancies, breast cancer in China shows aggressive behaviors. In patients with a large tumor size (>2–5 cm, accounting for 51.3% of cases), the mean age is in the 40s, which is a decade earlier than what is reported in Western countries [24,25]. Ultrasound-guided biopsies are more sensitive to detect breast cancers than stereotactic-guided ones in young women, especially for invasive carcinoma [26]. Further, the use of ultrasound in a screening setting is widespread in China. Ultrasound-guided CNB is the first choice for performing a percutaneous biopsy for most lesions seen or found to be highly suspicious on ultrasound. In China, 16G or 18G core needles are commonly used in most medical institutes. This is because in regards to mass lesions, target accuracy and proper procedures are essential for predicting the final results, regardless of the size of the core needle or the number of core samples [6]. Moreover, the smaller the needle diameter, the lower the friction will be with the surrounding tissues. Therefore, the puncture is easier to make and the strength of the shot is greater, which is helpful when the breast tissue is dense and causes difficulties in positioning the needle when the breast is relatively mobile. In addition, at least in theory, using small needles should decrease bleeding and pain [22]. The selection of a small gauge core needle may be reasonable from the viewpoint of needle handling, and the samples harvested by ultrasound-guided 16G or 18G CNB are approved by most pathologists and surgeons in our hospital. Then, if we assume that tissue samples obtained by 16G or 18G CNB could be adequate for an accurate diagnosis, why not use smaller needle for easier biopsy procedures and fewer complications?

In the present study, both 16G and 18G CNB had overall false-negative rates of 1.4%, which is acceptable compared with previously reported rates of 0% to 12% [13,16,20]. These results confirmed that ultrasound-guided 16G and 18G CNB are accurate and reliable alternatives to surgical biopsy for ultrasound-visible breast lesions.

However, a significant problem with CNB is the high rate of histological underestimation. A meta-analysis of underestimation of high-risk lesions in stereotactic-guided biopsy showed a rate of 40% [27]. Studies demonstrated that among lesions yielding DCIS at ultrasound-guided CNB, surgeries revealed infiltrating carcinoma in 16% to 55.5% of cases [21]. The rate of ADH underestimation with ultrasound-guided 14G CNB was even more highly variable (0% to 100%, mean 53%) [28,29]. Similar to these previous studies, the high-risk underestimation and DCIS underestimation rates of 16G and 18G CNB were also relatively high in the present study. Therefore, we recommend vacuum-assisted CNB or complete surgical excision of all such lesions detected by CNB, to ensure the detection of any coexisting malignancy or invasive cancer.

With the improvement of ultrasound resolution and contrast, microcalcifications contrasting with background hypoechoic areas or duct-like structures can now be detected by ultrasound [30]. It is very important to classify non-mass lesions visible using ultrasound because these lesions are more histologically heterogeneous than mass lesions, and usually include more DCIS and non-palpable lesions [6]. In the present study, there was a significant difference in the agreement between mass and non-mass lesions for 18G CNB. This indicates that for smaller caliber CNB performed on non-mass lesions, especially 18G, the diagnosis value is lower than biopsies using a larger core needle or vacuum-assisted CNB [31].

Small tumor size is proven to contribute to the inaccuracy of CNB [8]. In our study, the agreements decreased with smaller lesions. This finding is similar to a previous study performed using 14G CNB [8]. This may be the result of the poor lesion or needle visualization within the small lesion, influenced by partial volume effect artifacts towards the periphery of the lesion. Therefore, radiologists must be aware of these difficulties and should pay extra attention in lesions ≤10 mm when performing ultrasound-guided CNB. Larger caliber CNB or vacuum-assisted CNB is recommended.

There are certain limitations in the present study. First, lesions with benign results at CNB without subsequent surgical excision have been excluded. Therefore, a selection bias may exist, and it is possible that there were more false-negative diagnoses in the excluded cases. That is, the true sensitivity of CNB using 16G or 18G needles is equal to or lower than that calculated in this study. Maybe this is the reason why our false-negative rates are relative high compared with previous results (Table 4). Although the reports in Table 4 showed there were few missed malignant cases in the follow-up period (0 to 3 cases) [11-14], further study to follow patients with a benign CNB result who did not undergo excisional biopsy is necessary to know the actual sensitivities. Secondly, not every case was harvested with the same number of core samples, although a previous study indicated that two cores are sufficient to diagnose breast cancer, assuming that no technical error occurred [15]. Finally, this study did not make any direct comparison with 14G ultrasound-guided CNB. However, our data are compatible with data obtained using 14G CNB in previous studies [8-18].

**Table 4:** Published series of ultrasound-guided CNB

**Author**	**Year**	**Core needle size**	**No. malignant at CNB**	**False-negative rate**
Parker et al. [32]	1993	14G	34	0/34 (0%)
Smith et al. [10]	2001	14G	124	0/128 (0%)
Schoonjans et al. [11]	2001	14G	234	9/243 (3.7%)
Memarsadhegi et al. [12]	2003	14G	161	5/166 (3.0%)
Cystal et al. [13]	2005	14G	311	12/323 (3.7%)
Sauer et al. [14]	2005	14G	603	11/618 (1.8%)
Dillon et al. [33]	2005	14G	769	13/769 (1.7%)
Schueller et al. [16]	2008	14G	698	11/709 (1.6%)
Youk et al. [17]	2010	14G	1932	50/1982 (2.5%)
Povoski et al. [18]	2011	14G	386	8/386 (2.1%)
Present study		16G/18G	803	12/851 (1.4%)

## Conclusions

In conclusion, for ultrasound visible breast lesions, 16G and 18G ultrasound-guided CNB can be used as an accurate diagnostic alternative to surgical biopsy, at least in China. For mass lesions visible on ultrasound with a size >10 mm, 18G and 16G CNB are accurate and should be the first choice for ultrasound-guided CNB of these lesions. For mass lesions with a size ≤10 mm or for non-mass lesions, large caliber CNB or vacuum-assisted CNB is necessary. Considering the high rate of underestimation of high risk lesions by ultrasound-guided CNB, large volume vacuum-assisted biopsy or surgical excision is strongly recommended.

## Abbreviations

ADH: Atypical ductal hyperplasia; ALH: Atypical lobular hyperplasia; BI-RADS: Breast Imaging Reporting and Data System; CNB: Core needle biopsy; DCIS: Ductal carcinoma in situ; LCIS: Lobular carcinoma in situ.

## Competing interests

The authors declare that they have no competing interests.

## Authors’ contributions

JT and JZ participated in the conception and design of the study. YL, ZW and FL participated in the collection of the data. HQ and ML supported the study by assisting with the histopathological results analysis after surgical excision. JZ performed almost all the data analysis and drafted the manuscript. All authors read and approved the final manuscript.
